# Transcapsular Buttonholing of the Proximal Ulna as a Cause for Irreducible Pediatric Anterior Elbow Dislocation

**DOI:** 10.1155/2018/8986230

**Published:** 2018-05-09

**Authors:** Nick N. Patel, Robert W. Bruce

**Affiliations:** Department of Orthopaedic Surgery, Emory University School of Medicine, 59 Executive Park S, Atlanta, GA 30329, USA

## Abstract

Anterior elbow dislocations in the pediatric population represent rare and sometimes difficult injuries to manage. Associated olecranon fractures are even more uncommon with limited literature existing on the topic. We present the case of a six-year-old male with a traumatic transolecranon anterior elbow fracture dislocation in whom closed reduction was prevented by buttonholing of the proximal ulna through the anterior joint capsule. This case of pediatric anterior elbow fracture dislocation provides insight into an uncommon and challenging injury complex.

## 1. Introduction

Traumatic elbow dislocations in the pediatric population are relatively uncommon and have been estimated to represent between 3–6% of all pediatric elbow injuries [1]. Posterior dislocations comprise the vast majority of these with anterior elbow dislocations representing only a very small subset. Anterior elbow dislocation with associated olecranon fracture in skeletally immature individuals has only been described in about 10 case reports in the literature to our knowledge [2–5]. Furthermore, we were only able to identify one case report in the literature regarding a pediatric transolecranon fracture dislocation in which closed reduction was precluded by entrapment of the anterior capsule [5]. We aim to add to the current literature by presenting the case of a skeletally immature patient with a traumatic anterior elbow dislocation with associated olecranon and radial head fractures. Closed reduction in this case was prohibited by buttonholing of the proximal ulna through the anterior joint capsule.

## 2. Case Presentation

**Figure 1:**
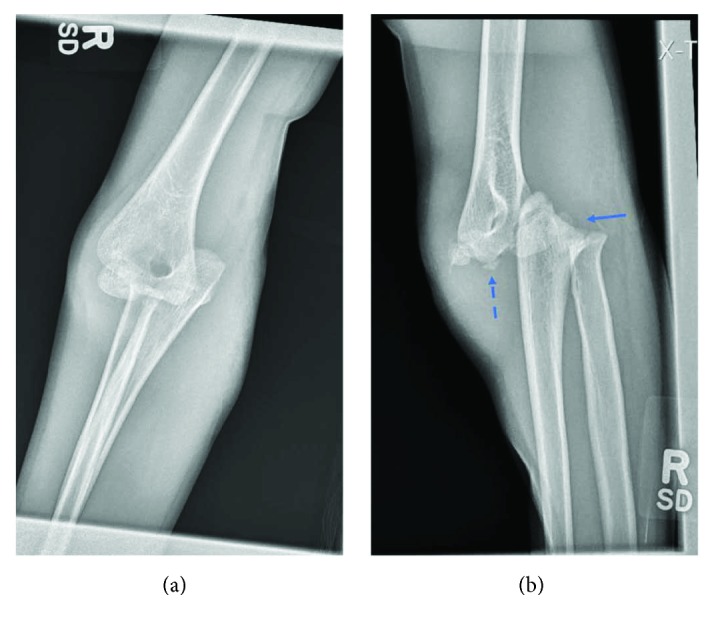
AP (a) and attempted lateral (b) radiographs of the right elbow demonstrating anteromedial elbow dislocation in a skeletally immature individual. The solid arrow points to the Salter-Harris radial head fracture with significant radial head displacement. The dashed arrow points to an ossific fragment near the distal humerus which likely represents the olecranon avulsion.

**Figure 2:**
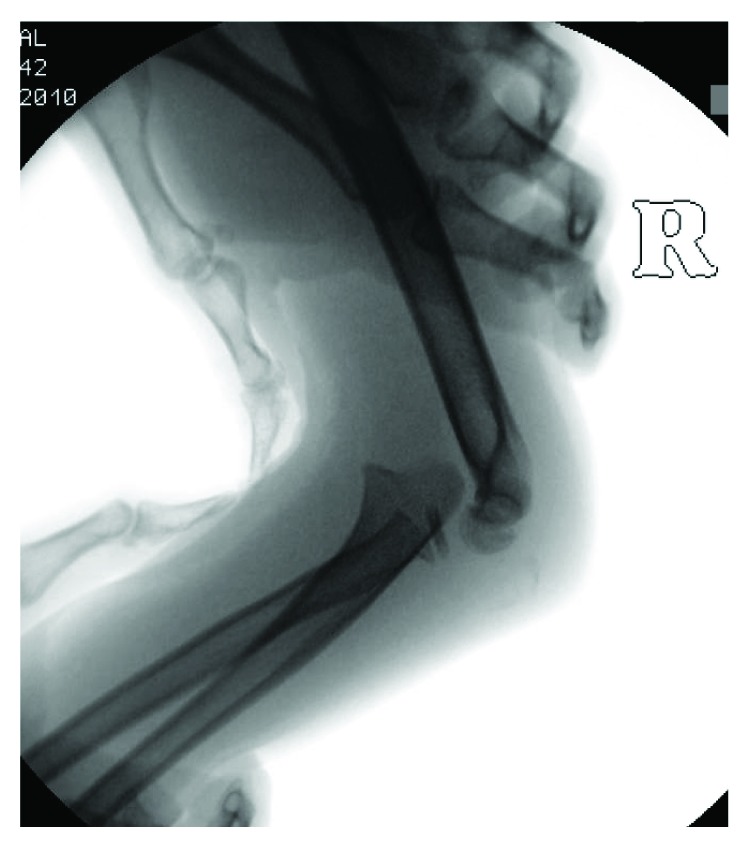
Lateral radiograph of the right elbow showing continued anterior dislocation of the ulnohumeral joint despite attempted reduction. The Salter-Harris II fracture of the radial head can again be noted.

**Figure 3:**
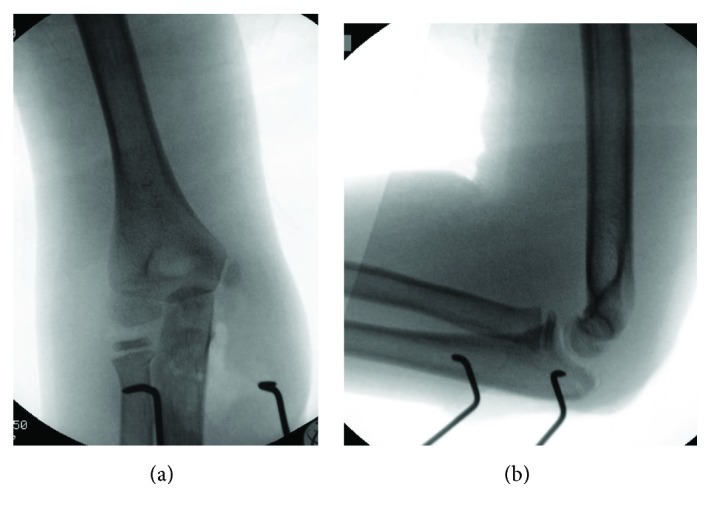
AP (a) and lateral (b) radiographs of the right elbow demonstrating anatomic reduction of both the ulnohumeral and radiocapitellar joints. The radial head fracture has improved alignment.

**Figure 4:**
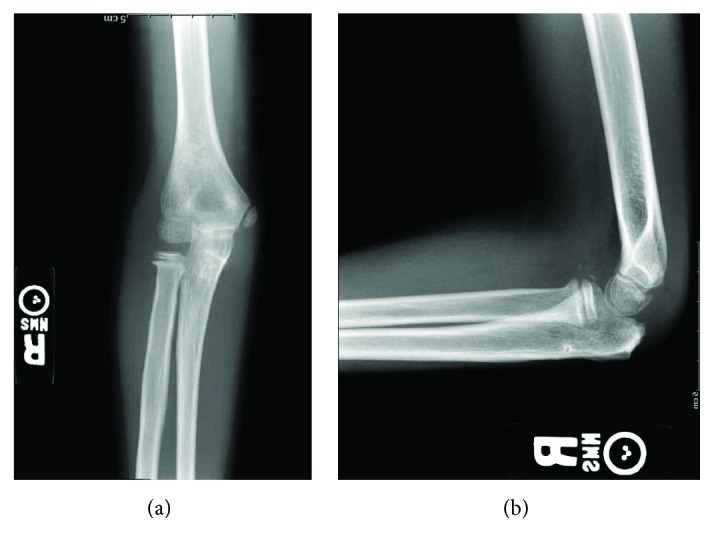
AP (a) and lateral (b) radiographs of the right elbow 7 months postoperatively demonstrating continued anatomic reduction of the ulnohumeral and radiocapitellar joints. The radial head and olecranon fractures are well healed. The patient's physes remain open.

The patient in this case was an otherwise healthy 6-year-old male who presented to the emergency room with a right elbow deformity from an injury sustained while playing football. He reported being tackled and falling back, leading to a direct impact onto his flexed right elbow. Examination demonstrated his right arm to be neurovascularly intact despite having a closed gross deformity at the elbow joint. X-ray evaluation revealed an anteromedial elbow dislocation with fracture of the radial head (Figure 1). Two attempts were made at closed reduction in the emergency room under ketamine sedation with no appreciable reduction or improvement in alignment. Given the lack of success with closed reduction, the decision was made to take the patient to the operating room urgently for closed versus open reduction and possible fixation. Once under general anesthesia and with muscle relaxation employed, another closed reduction maneuver was attempted again with no success (Figure 2). A direct posterior approach to the elbow was then performed with exposure carried around to the subcutaneous border of the ulna. It was noted that the olecranon apophysis remained in its anatomic position despite the ulna being anteriorly dislocated. Interestingly, the proximal ulna was found to be buttonholed through the anterior joint capsule, thus preventing reduction of the joint. The radial head was also noted to have an angulated Salter-Harris II fracture which was realigned with direct pressure application. No epicondylar fracture was noted with direct inspection. Once the entrapped proximal ulna was freed from the anterior capsule, it was easily reduced into its anatomic position (Figure 3). Braided suture was used to reattach the olecranon apophysis to the proximal ulna through two drill holes in a figure of eight fashion. Final clinical exam and fluoroscopy images through a full range of motion revealed stable reduction of both the elbow joint and olecranon apophysis. The patient was splinted for approximately four weeks followed by progression of gentle passive and active elbow range of motion. At his most recent 7-month follow-up, the patient was doing very well with 0^o^–135^o^ of elbow extension and flexion and 75^o^ each of forearm pronation and supination. Radiographs revealed well aligned and healed fractures with maintained physes (Figure 4).

## 3. Discussion

A common mechanism for anterior elbow dislocation is thought to be a direct blow to the posterior joint as occurred in this particular case. With this injury mechanism and resulting deformity, it is important for clinicians to be cognizant of a likely associated olecranon fracture [6]. While it was not obvious on preoperative radiographs in our case, operative exploration did reveal an avulsion injury of the olecranon apophysis. Studies have shown that the olecranon ossification center generally appears between 9-10 years of age; therefore, it can be challenging to radiographically identify olecranon injuries at the osseocartilaginous junction prior to this age [7]. The olecranon physeal injury in this situation was treated with a tension band construct using braided suture. This was noted to provide good reduction and clinical stability.

Multiple closed reduction attempts were unsuccessful in this patient due to the proximal ulna being buttonholed through the anterior joint capsule. This is not a well-known occurrence, and to the best of our knowledge, only one prior case report of this exists [5]. Relief of this entrapment allowed for easy ulnohumeral joint reduction. When evaluating patients with persistent irreducible anterior elbow dislocations, this etiology should be considered. Anterior elbow fracture dislocations in the pediatric population are rare but challenging presentations that are not well characterized in the literature. This presented case illustrates some unique features which we feel add to the current limited knowledge surrounding the topic.
